# Supplementary material for: Natural hybridization in heliconiine butterflies: the species boundary as a continuum
Source: BMC Evol Biol. 2007 Feb 23;7:28. doi: 10.1186/1471-2148-7-28 (PMC1821009; doi:10.1186/1471-2148-7-28)
Supplement: Additional File 1 — Hybrids between species of Heliconius and Eueides butterflies: a database. HTML file linking to database of all known wild-caught interspecific hybrid specimens in the Heliconiina, consisting of introductory text, a list of specimens, together with collection data and photographs of the specimens, and links to information about some artificial hybrids and mutants in the group. This is an edited copy of our online database of Heliconius hybrids [102]. To view database, download zip file and extract to a separate folder, then open index.html within that folder. [file 1471-2148-7-28-S1.zip › hecele03.html]

hybrid hecele03


---


  
Hybrid between *Heliconius hecale zeus* and *H. elevatus perchlorus*
  
Bolivia
  
© Andrew V.Z. Brower

Return to table of hybrids

To next hybrid
  
To previous hybrid

```
NOTES

No:                      16
Genus of species 1:      Heliconius
Species 1:               hecale
Subspecies of species 1: zeus
Genus of species 2:      Heliconius
Species 2:               elevatus
Subspecies of species 2: perchlorus
Sex:                     m
Country:                 Bolivia
Locality:                Chapar�: Todos Santos
Year:                    1969
Photo no.:               hecele03
Named hybrid:
Collection:              Wien
Collector:               H. Holzinger
Author/publication:
Notes:                   F1? Andrew Brower (pers. comm., 1 July 2000): 'I think it is pretty clearly hecale x elevatus'
```

**Last updated:** 18 October 2003

---
